# Perceptions and insights: A qualitative assessment of an AI-assisted psychiatric triage system implemented in an outpatient hospital setting

**DOI:** 10.1177/20552076251384835

**Published:** 2025-10-15

**Authors:** Oleksandr Knyahnytskyi, Jazmin Eadie, Kimia Asadpour, Callum Stephenson, Megan Yang, Taras Reshetukha, Christina Moi, Tricia Barrett, Meghanne Hicks, Gilmar Gutierrez-Nunez, Anchan Kumar, Jasleen Jagayat, Saad Sajid, Charmy Patel, Christina Holmes, Amir Shirazi, Vedat Verter, Claudio Soares, Mohsen Omrani, Nazanin Alavi

**Affiliations:** 1 Department of Psychiatry, Faculty of Health Sciences, 12363 Queen’s University, Kingston, Canada; 2 Centre for Neuroscience Studies, 98617 Faculty of Health Sciences, Queen's University, Kingston, Canada; 3 OPTT Health, Toronto, Canada; 4 71459 Kingston Health Sciences Centre, Kingston, Canada; 5 Smith School of Business, 120458 Queen's University, Kingston, Canada

**Keywords:** Online cognitive behavioral therapy, mental health, artificial intelligence, psychiatry, psychotherapy, cognitive behavioral therapy, qualitative, perceptions and experiences

## Abstract

**Introduction:**

The Canadian healthcare system is approaching a breaking point. With mental health being a leading cause of disability, innovative solutions are necessary to provide adequate care. Digital mental health programs, such as electronic cognitive behavioral therapy, have proven effective in reducing the challenges of traditional psychotherapy, such as long waitlists, stigma, geographic barriers, and time constraints. Furthermore, artificial intelligence (AI) has shown potential utility within the healthcare system, particularly in treatment recommendations and improving patient engagement. Despite the benefits that AI and digital mental health programs provide, they are rarely implemented in real-world healthcare settings.

**Objective:**

This study aims to explore patient experiences and perceptions of an AI-assisted triage system paired with a digital psychotherapy program. The objective is to highlight the potential modifiable barriers to implementing these digital systems in real-world healthcare settings.

**Methods:**

Forty-five adult outpatient psychiatry patients (*n* = 45) who used an AI-assisted triaging system and digital psychotherapy modules were surveyed through Qualtrics. This survey examined their perceptions of AI within mental healthcare, its utility within triaging, and their experiences with the digital psychotherapy program. Free-text survey responses were independently coded and analyzed using thematic analysis.

**Results:**

Thematic analysis revealed three major themes for clients’ perceptions of the AI-assisted triage system: (1) AI as a replacement, (2) the utility of AI, and (3) AI complexity recognition. For the digital psychotherapy program, the themes were: (1) interactions with technology, (2) online therapy program structure, and (3) differential user experience.

**Conclusion:**

Participants highlighted the importance of human oversight to ensure accuracy and liked that the AI allowed them to access care faster. Suggestions for improving the digital psychotherapy program included enhancing user-friendliness, increasing human contact, and making it more accessible for neurodivergent individuals.

## Introduction

### Background information

Access to timely and effective mental healthcare remains a significant challenge in Canada. Despite general improvements in the quality of care over the years, long waitlists, provider shortages, and systemic inefficiencies leave many patients with unmet healthcare needs.^1–7^ These concerns, and the lack of readily available resources, calls for new and innovative solutions. Digital mental health interventions, such as online cognitive behavioral therapy (eCBT), have emerged as scalable tools to address these gaps by making healthcare services more accessible, helping to streamline the workflow of hospitals and increasing access to care.^8,9^

eCBT involves structured, evidence-based therapy modules delivered through online platforms, allowing individuals to engage in cognitive and behavioral strategies to manage mental health challenges.^10^ eCBT specifically addresses concerns of accessibility, as it helps overcome barriers such as geographic limitations, long wait times, and stigma associated with seeking in-person care.^11^ Additionally, eCBT minimizes the time needed for direct clinician involvement, which in turn helps reduce the financial burden on the patients and alleviates pressure on overburdened mental health services, helping address systemic inefficiencies.^10,12^ While digital interventions help reduce some structural barriers (e.g. geography, scheduling, stigma), they do not inherently resolve the bottleneck in initial patient triage. However, with the digital nature of interventions such as eCBT, it becomes an ideal candidate for integrating more novel and advanced technologies, such as artificial intelligence (AI), to help further optimize care pathways.^13–15^

In mental healthcare, AI-assisted tools have been integrated into diverse applications, such as symptom monitoring apps that use natural language processing to detect early signs of relapse, chatbots delivering cognitive behavioral therapy, and predictive models that flag high-risk patients in crisis.^16–18^ These innovations are reshaping care pathways, yet their success depends not only on technical accuracy but also on the quality of the interaction between the patient and the digital system. This emerging construct, referred to as the *digital therapeutic alliance (DTA)*, reflects the patient's perception of the digital tool as a trustworthy, responsive, and supportive therapeutic partner.^19^ A strong alliance in digital interventions has been associated with higher engagement, lower dropout rates, and improved treatment outcomes.^20,21^ Understanding how patients experience this alliance in real-world psychiatric outpatient settings is therefore crucial, as perceptions of “relational connection” and system responsiveness may influence both acceptability and clinical effectiveness of AI-based mental health tools.

In recent years, AI has seen a notable increase in utility within healthcare settings due to its effectiveness in tasks such as treatment recommendations, patient engagement, clinical decision support, diagnostic pattern recognition, symptom classification, resource allocation, treatment adherence, and therapy effectiveness.^8,13^ Applied to mental health triage, AI can analyze narrative data to help identify symptom severity and recommend appropriate care pathways, thus improving both efficiency and equity in service delivery. Previous research has demonstrated that patient engagement and sustained use of digital mental health interventions are closely linked to user perceptions of accessibility, personalization, and therapeutic alliance.^19,21^ In psychiatric outpatient settings specifically, studies evaluating eCBT and other online interventions have reported that positive perceptions of program relevance, ease of use, and interpersonal connection can improve adherence and reduce dropout.^22^ Conversely, perceptions of low personalization, insufficient human contact, or technological barriers have been associated with reduced engagement.^23,24^ Despite this growing evidence, there remains a scarcity of qualitative research exploring these dynamics in real-world psychiatric outpatient contexts, particularly for programs integrating AI-assisted triage with digital psychotherapy. Understanding these experiences is crucial because user perceptions directly influence treatment engagement, satisfaction, and adherence in digital care settings. Understanding these perspectives allows for the refinement of intervention design, the enhancement of therapeutic alliance in digital contexts, and the identification of barriers that may otherwise limit the effectiveness and scalability of such programs in psychiatric outpatient care.

In the current study, we conducted a qualitative exploration of the personal experiences of individuals who engaged with the AI-assisted triage system and subsequent digital psychotherapy program developed and implemented by the Queens University Online Psychotherapy Lab (QUOPL) as part of a quality improvement (QI) study. The AI-assisted triage tool was developed by the QUOPL in collaboration with the study team and implemented on the secure OPTT platform. It combined natural language processing (NLP) algorithms with supervised machine learning models trained on anonymized patient narratives and validated symptom questionnaires. Upon referral to the program, participants completed an online triage module that included free-text narratives about their mental health experiences, along with structured screening measures. The AI analyzed these inputs to identify linguistic and symptom patterns, assigning a preliminary severity category (mild, moderate, severe), recommending an urgency level for psychiatric consultation, and suggesting an appropriate care pathway (e.g. online psychotherapy alone, online psychotherapy with therapist check-ins, or online psychotherapy with psychiatrist involvement).

Importantly, the AI tool did not make diagnostic decisions or initiate treatment independently. All recommendations were reviewed and confirmed by a licensed psychiatrist before being communicated to patients or enacted in care. Patients were shown their recommended care pathway and urgency level, but not the full algorithmic rationale behind the decision, which may have influenced perceptions of transparency. This system was integrated into the existing intake process at Kingston Health Sciences Centre (KHSC), enabling clinicians to prioritize higher-acuity patients while offering timely, structured interventions to those in lower-acuity categories.

By analyzing free-text survey responses, we highlight modifiable barriers and enablers to implementations, with implications for improving user experiences, therapeutic engagement, and trust in digital care pathways.

## Materials and methods

This study has received ethical approval from the Queen's University Health Sciences and Affiliated Teaching Hospitals Research Ethics Board. This study was conducted as an independent part of a QI study focused on the implementation of an AI-triage system at Hotel Dieu Hospital, Kingston, ON, Canada. The current qualitative study focused on the use of survey data, utilizing free-text responses as the primary method of data collection. The open-ended survey format offered participants flexibility to share experiences in their own words and at their own pace, potentially enabling richer personal reflections than closed-response formats. However, open-ended survey responses also present constraints on interpretive depth, as they do not allow for in-the-moment probing, clarification, or follow-up questions that can enrich qualitative insights. These affordances and limitations should be considered when interpreting the thematic findings presented in this study. Participants were provided access to a survey, which they could choose to fill out either at the end of the paired AI-eCBT program or upon drop out from said program. As completion of the surveys was entirely optional, implied consent was obtained. Surveys were all anonymous, and any potentially identifying information was anonymized during the process of data collection and organization. The use of free-text responses allowed participants to share a more detailed and personalized view of their experiences with the AI-triage system they interacted with, providing more transparency regarding their experiences.

### Participants and study design

A total of 101 patients participated in our QI study implemented at KHSC from October 2023 to March 2024. Participants were adult patients (≥18 years of age) who had been referred by family physicians to the ambulatory clinic at KHSC, HDH in Kingston ON. Patients on the waitlist, referred between January and September 2023, were invited to participate in the study utilizing an AI-assisted triage tool paired with digital psychotherapy modules. Upon completion or dropout from the initial study, participants were offered the option to complete an anonymous Qualtrics survey outlining their thoughts and experiences with both the AI-assisted triage and the digital psychotherapy program. Of the 101 individuals who participated in the QI study, 45 voluntarily completed the post-intervention Qualtrics survey. These 45 participants form the qualitative sample for the present analysis. The survey was optional and made available to participants following their completion or discontinuation of the intervention.

The AI-assisted triage tool integrated NLP techniques and supervised machine learning models trained on anonymized clinical narratives and validated symptom measures. The system analyzed participants’ written accounts and questionnaire responses to estimate symptom severity (categorized as mild, moderate, or severe), recommend urgency levels for psychiatric assessment, and suggest tailored care pathways (e.g. eCBT alone, eCBT with therapist check-ins, or eCBT with psychiatrist consultation). These recommendations were reviewed and confirmed by a psychiatrist before implementation. Patients were shown their recommended care pathway, but the underlying algorithmic reasoning was not directly displayed, which may have influenced their perceptions of transparency.

**Figure 1.**
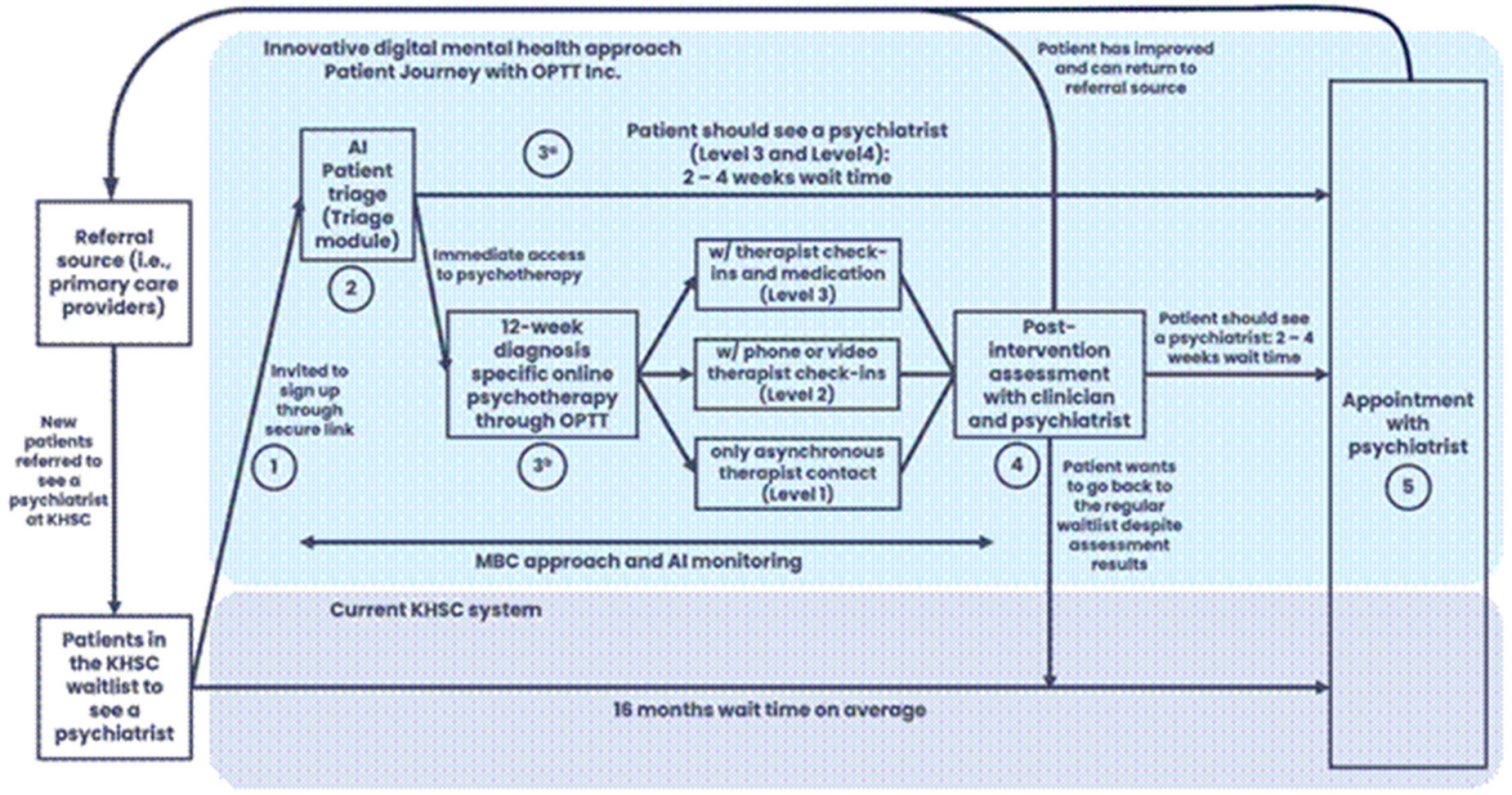
Patient journey as part of the QI study conducted by our team at QUOPL. The figure outlines (1) the patient journey in the current triage system at KHSC and (2) the patient journey through the AI-triage approach.

*Note:* QI: quality improvement; QUOPL: Queens University Online Psychotherapy Lab; KHSC: Kingston Health Sciences Centre; AI: artificial intelligence.

As shown in Figure 1, outlining the patient journey, the triage tool assessed each patient's needs, suggesting a level of care (mild, moderate, or severe) and determined the urgency and necessity of seeing a psychiatrist. It also recommended suitable digital psychotherapy options and the level of support required in psychotherapy, such as fully online sessions, online therapy with weekly therapist check-ins, or online therapy with weekly check-ins and psychiatrist appointments. All AI-assisted assessments were finalized by a practicing psychiatrist to ensure accuracy.

The eCBT program delivered through the OPTT platform consisted of 8–12 weekly, asynchronous modules based on structured cognitive behavioral therapy principles targeting disorder-specific (e.g. depression, anxiety, posttraumatic stress disorder, obsessive-compulsive disorder, etc.). Each module included psychoeducational content, interactive exercises, and structured homework assignments, with a focus on cognitive restructuring, behavioral activation, and skills training. Content was presented in a narrative format using relatable fictional characters to enhance engagement and relatability. Participants submitted weekly homework assignments, after which they received tailored written feedback from a trained therapist. Depending on their triage level, participants received either (1) eCBT only, (2) eCBT with weekly phone-based check-in calls, or (3) eCBT with both phone check-ins and psychiatrist consultation. Modules were self-paced within weekly deadlines, and automated reminders were used to support adherence.

### Intervention

For this study, 45 adult participants (*n* = 45, 18 years old and above) directly involved in the use of the AI-triage system received an anonymous Qualtrics survey link through an email address they had previously provided. Before beginning the questionnaire, the survey's purpose and procedures were clearly explained, and participants reconfirmed their consent to proceed. Ethical approval for this study was obtained from the Queen's University Health Sciences and Affiliated Teaching Hospitals Research Ethics Board in Kingston, ON, Canada.^20^

### Survey development

The postparticipation survey was designed based on the framework of previous surveys implemented by the research team to capture the user experiences of participants taking part in AI-assisted triage and the digital psychotherapy program.^25^ The content was created through comprehensive literature reviews and validated qualitative assessment tools. Before distribution to the participants, the survey underwent several rounds of revision by team members with expertise in AI and online psychotherapy. Although the survey was not formally validated, the survey was reviewed by experts in digital psychotherapy and pilot tested internally.

### Data analysis

The postparticipation survey was created using Qualtrics.^26^ Inductive thematic analysis was used to investigate the data collected from the survey responses. Thematic analysis is a common qualitative methodology used to identify and interpret major patterns within a text-based data set.^27^ The use of thematic analysis allows for the emergence of themes or “patterned responses” from within a data set, which in turn can help guide our understanding of complex phenomena such as patient perceptions and experiences.^28^ To provide greater transparency, our analysis followed the six phases of reflexive thematic analysis^28^ First, two researchers (OK and JE) independently reviewed all participant responses to familiarize themselves with the dataset and note initial impressions. Second, codes were developed through inductive, line-by-line coding without using a predefined codebook, allowing codes to emerge from the data. Researchers met to discuss their initial codes to informally assess coding consistency. Discussions also involved the researchers’ reflection on personal biases and experiences in regard to code development, in which researchers explained their coding patterns. Themes were developed through iterative analysis in which researchers independently grouped codes into themes and then engaged in collaborative discussions with other researchers in order to compare their interpretations. Discrepancies were resolved by reiteratively applying and refining the independently developed themes. Themes were reviewed to ensure they accurately represented participant narratives, and subthemes were collaboratively identified where appropriate. Fifth, each theme was defined and named to capture its core meaning, with a focus on preserving the nuance of the qualitative accounts. Finally, once themes were established, researchers reviewed all of the coded reference text and collaboratively decided on the most representative quotations aligning with the themes and subthemes. Reflexive discussions throughout the process considered the research team's positionality. More specifically, in the process of developing the codes and themes, researchers reflected on their prior experiences with digital mental health platforms, artificial intelligence-assisted health tools and overall interest in improving access for underserved populations with emphasis on how these experiences may have impacted their interpretations. While some codes reflected relational aspects (e.g. human contact, interpersonal connection) and others focused on system responsiveness or personalization, these elements occasionally appeared together within the same participant narrative. We chose to retain them together in such instances to preserve contextual nuance, while clarifying in the subtheme introductions whether the emphasis was on relational or system-related aspects. Data was collected and analyzed until data saturation was reached

### Methodological rigor and quality assurance

To ensure the rigor, credibility, and transparency of our qualitative analysis, we adopted multiple strategies aligned with the Consolidated Criteria for Reporting Qualitative Research framework, as outlined below. Please see.

#### Coding procedures

An inductive thematic analysis approach was employed without a prespecified formal codebook. Research personnel independently reviewed all participant responses to familiarize themselves with the data, generated initial codes and themes based on emergent content, and then collaboratively refined these interpretations. Discrepancies between coders were discussed and resolved through consensus, promoting analytic rigor. Although formal intercoder reliability metrics (e.g. Cohen's κ) were not calculated, consistency was strengthened through regular meetings, iterative drafts, and collective review of coding decisions.

#### Credibility

Credibility was reinforced by using multiple analysts in the coding process (investigator triangulation) and by promoting reflexive discussions among coders regarding interpretations of participant responses. Themes were refined collaboratively to ensure they remained grounded in the data rather than shaped by preconceptions.

#### Transferability

Transferability was supported by providing rich descriptions of the clinical context, participant demographics, and the intervention (AI-assisted triage system and digital psychotherapy program). This contextualization allows readers to assess the potential applicability of the findings to other settings or populations.

#### Dependability

Dependability was established through careful documentation of analytic decisions across the coding process. Although no formal audit trail was created, analytic decisions were naturally recorded through successive drafts, version control of coding frameworks, and team discussions, ensuring a transparent evolution of themes.

#### Confirmability

Confirmability was strengthened by grounding all findings explicitly in participant quotations, minimizing the influence of researcher bias. Reflexive discussions about potential preconceptions, especially given some researchers’ involvement in the AI system's development, further supported the objectivity of the interpretation.

#### Data saturation

This study did not use a formal sample size or power calculation, as it employed a qualitative methodology within a QI framework. The sample consisted of 45 adult participants who had completed or withdrawn from an AI-assisted triage and digital psychotherapy program at a hospital-affiliated outpatient clinic. The sample size was determined based on feasibility and the goal of achieving thematic saturation, which was monitored throughout analysis and considered achieved after approximately 40 responses, at which point no new substantive themes were identified. This suggests that the sample size was sufficient to capture a comprehensive range of participant perspectives.

## Reflexivity and positionality

The primary analysts of this study (OK—he/him and JE—she/her) are affiliated with the QUOPL and have experience as care providers using digital mental health platforms, including OPTT, in separate clinical settings. While neither author holds a direct affiliation with the specific AI triage tool evaluated in this study, both have worked on research projects exploring the accessibility, efficacy, and delivery of digital mental healthcare. Their familiarity with digital platforms and interest in improving access to care for underserved populations inform an openness to innovation, but also a critical awareness of the limitations of technology in clinical contexts. To mitigate potential biases, the authors engaged in independent coding, iterative theme development, and reflexive team discussions throughout the analysis process. All interpretations were grounded in participant data, and analytic decisions were guided by consensus to ensure methodological transparency and trustworthiness.

In practice, our positionality shaped our interpretations in several ways. For example, familiarity with the practical constraints of implementing digital interventions allowed us to recognize the operational feasibility of some participant suggestions, while lived experience as care providers heightened our sensitivity to relational and trust-related concerns. To mitigate bias, we deliberately sought to challenge our interpretations during team discussions, invited feedback from coauthors not directly involved in coding, and ensured that final themes were substantiated by multiple, direct quotations.

## Results

Data extracted from the Qualtrics survey that examined patient perceptions of the AI-assisted triage and eCBT program revealed six themes. Three themes related to the use of an AI-assisted triage system: (1) Mixed Views on AI's Role in Mental Healthcare, (2) Utility of AI, and (3) AI Complexity Recognition emerged. Additionally, there were three themes related to the eCBT program: (1) Interactions with Technology, (2) Online Therapy Program Structure, and (3) Differential User Experience. Thematic analysis was conducted using a reflexive approach, which prioritizes the depth, nuance, and contextual meaning of participant narratives over the frequency of theme occurrence. Therefore, we do not report the number of participants per theme or include a summary table, as this could imply a level of quantification inconsistent with our analytic aims. No new themes emerged after analyzing the responses from the 40th participant, suggesting thematic saturation was achieved.

While the themes presented reflect patterns identified through iterative analysis, not all views were universally held. Thematic representation aimed to capture a spectrum of perspectives, including both common sentiments and more divergent or critical viewpoints. The themes and their associated subthemes are described in more detail below.

### Participant experiences and perceptions of the AI-assisted triage

#### Theme: Mixed views on AI's role in mental healthcare

##### Subtheme: Need for and potential of AI in healthcare

Participants expressed diverse opinions regarding the implementation of AI-assisted systems within real-world healthcare settings. Many participants supported the use of such a system, focusing on its utility as a gateway towards accessible care: “*Whatever tool helps connect mental health professionals with patients in need is all goodness.”* Another participant shared a similar sentiment, emphasizing the necessity of AI due to deficiencies in our current healthcare systems: “*It's [AI] necessary at this point since there are not enough care providers.”*

Conversely, a smaller subset of participants expressed concerns that AI may not be well-suited to capture the complexity of mental health care: *“AI is improving, but humans are complicated; therefore, AI is invalid to treat real people.”* These concerns reflect the nuanced and sometimes contradictory views expressed in the dataset, rather than a singular or dominant perspective

##### Subtheme: Need for human oversight

Relating to the need for and potential of AI, participants highlighted a need for human oversight in the implementation of an AI-assisted healthcare system. One patient mentioned the importance of human oversight in monitoring the behavior of the system:

I work in AI, and I think that it can be very beneficial in health care, but it is crucial to have a human in the loop and to monitor the behaviour of the systems consistently.

Additionally, other participants discussed the necessity of human control over diagnosis and medication prescription: “*It [AI] has potential, but there has to be access to somebody who can diagnose and prescribe medications.”* When discussing the differences between traditional and AI-assisted triage, one participant mentioned that the AI-assisted triage system was “*Ok - but it can miss that ‘gut’ feeling & anyone or anything that fits outside the box.”*

#### Theme: Applicability of AI

##### Subtheme: Utility

Participants generally supported the use of AI to improve the efficiency of the care provided in a healthcare setting. “I believe efficiency in the service of fast-tracking patients to different forms of care is important, and if AI helps with that, then I have no problem with it.” When discussing the differences between the care the participants have previously received and the AI-assisted system, one participant mentioned, “I could not access a psychiatrist through my family doctor, so this intake trial was an enabler for me.” Additionally, another participant mentioned that one of the benefits of the system was that “I got the help and medication I needed after 37 years.” In contrast, some participants were not as open to the application of AI highlighting a limited acceptability of its utility: “For intake, I would accept AI, but for diagnosis or ongoing treatment, I would NOT support AI at this time.”

##### Subtheme: Perception of connection to AI-assisted tools

This subtheme focuses on participants’ perceptions of relational closeness or distance in the AI-assisted triage process, as distinct from the system's adaptability or personalization. When it came to discussing the key limitations of the AI-assisted triage system, some participants discussed a lack of personal connection as being a key constraint in their acceptability of the use of AI: “*I do not feel anyone can get a truly deep connection with an AI facilitated healthcare program - it was not for me.”* However, participants mentioned this in a more general sense regarding their views of AI in healthcare as a whole: “*When care and social interactions are compromised, I disagree with having AI in healthcare. Though, when it is used positively in [this] way, I felt it was used in this program.*

#### Theme: AI complexity recognition

##### Subtheme: Gaps in AI's clinical utility

In general, participants felt that AI is not able to account for the complexity of mental illness to the same degree as a human. As a result, this might have led to unaddressed needs in some of the participants.

A small subset of participants raised concerns regarding their unaddressed needs relating to neurodiversity. In this context, participants used the term “neurodivergence” in reference to more specific conditions such as attention deficit hyperactivity disorder (ADHD). Concerns raised were related to the program's ability to differentiate between neurodivergence symptoms and psychiatric symptoms, suggesting that neurodivergence needs might have gone unaddressed with the digital psychotherapy program. Participants mentioned simply that, “*Neurodivergence has gone unaddressed,”* or as another participant discussed in more detail: “*I am concerned for others that the AI diagnosed me with depression when my real problem is ADHD.”*

Though participants were generally open to AI, many discussed various limitations to its use, highlighting human complexity, the lack of specialized programs, and unmet needs as reasons that AI was not a favorable alternative.

Okay- but it can miss that ‘gut’ feeling & anyone or anything that fits outside the box.

AI is improving, but humans are complicated, therefore, AI is invalid to treat real people.

Finally, one participant mentioned a discrepancy between care needs, and the speed of care when answering a question on whether they found the speed of the AI-assisted triage to be better or worse than traditional methods. “*I am not sure the speed is relevant. I just do not see how a quicker service would be of use if the service is not addressing my needs.”*

### Participant experiences and perceptions of the digital psychotherapy program

#### Theme: Interactions with technology

##### Subtheme: Problems with the technology

Participants expressed various frustrations and challenges regarding the user experience with the platform. Common issues included difficulties logging in, an inability to access or read feedback, receiving incorrect modules, and not having an “*intuitive user design.”*

There are a lot of kinks in the system that need to be worked out to keep users from getting frustrated. I experienced login issues, and I found some of the User Experience challenging to understand at times.

My biggest issue was with the technical problems. For someone like me, problems like not being able to read the feedback or being given the wrong module are enough to derail the momentum and gave me a very convenient excuse to give up.

#### Theme: Online therapy program structure

##### Subtheme: Convenience, flexibility, diversity, and accessibility

Clients expressed mixed feelings about the program's level of convenience, flexibility, and accessibility. Some appreciated the ability to express themselves privately without the immediate presence of a care provider, commenting that this feature provided an outlet and access to care while they waited to see a psychiatrist.

I like it; the fact that the professional is not there looking at you, and you can just write your heart out without having to worry about social cues is useful.

I think it is great that people have an outlet while waiting for a psychiatrist.

##### Subtheme: Program repetitiveness and strictness

Some participants found the content of the program to be repetitive. Others found the deadlines to be challenging, which resulted in them finding the program to be more of a hindrance, resulting in various negative feelings such as anger.

Repetitive info

More of a hindrance than helpful.

This program was frustrating. Between deadlines, difficulties navigating the online program & the repetitiveness, I do not recommend it. It made me more upset and angrier than I had before attempting the program in the first place.

#### Theme: Differential user experience

##### Subtheme: Level of helpfulness

In addition to relational support, several participants evaluated the program's helpfulness based on how personalized and relevant the feedback felt to their needs. Differing experiences occurred for participants in this program, with some being helped and others feeling like this program was a hindrance. Clients who found this program to be helpful enjoyed the feedback and found benefits in the ability to continually set goals. Some individuals commented on having received the care that they had been seeking for years.

I got the help and medication that I needed after 37 years.

Feedback was helpful.

I found it beneficial to have set goals every week, and the feedback was reassuring. I found it challenging to remember all the content, there should be reminders in each session of the last session to reinforce the content.

In contrast, other participants felt like they were in the same place that they were in before starting the program. As a result of the therapeutic relationship, a participant expressed feeling like they needed to make stuff up to fit the homework.

I had the hope that when I was done with the program, I would be connected to a psychiatrist right away so I could continue the treatment. But now I am kind of back to where I was at the beginning. Having to go see a doctor at a walk-in clinic and hope that what they give me will work.

A few weeks into it, it became a task I needed to do to please the person calling me once a week. The experience was a bit like an educational course with due dates and assignments. I was completing the assignments to please the person calling me (like I was trying to get a good grade). I found myself making up things or thinking back into my past to come up with things that fit the assignments. I finally realized that completing assignments to please the person calling me is not what I should be doing to deal with and work through the issues I am trying to work through. This decision to quit the program has nothing to do with the person calling me. It is the program and how it is being presented.

##### Subtheme: Perceptions of the need for human contact

This subtheme addresses the relational and interpersonal dimension of participants’ experiences with the eCBT program, emphasizing voice-to-voice or in-person contact rather than technological features. Throughout the program, each participant was supported by a care provider who worked with them in various capacities. Everyone received feedback; some received weekly phone calls, and others got both of those and saw a psychiatrist. Participants had varying reactions to different levels of care. Some felt like the care provider kept them on track and appreciated how the weekly phone calls could clarify any confusing content from the eCBT. However, a common theme was that people who only received eCBT tended to feel like they missed out on the back-and-forth aspect of verbal communication.

I appreciated that the [care provider] was there and kept me on track. If there had not been that human element, I certainly would not have finished.

All contact with people was great.

Great doctors.

The benefits of having a knowledgeable mental health expert are unparalleled. Any issues I had understanding the online portions were cleared up during the weekly phone call.

Talking with someone behind a screen is not personal, and it is hard to communicate your true struggles this way.

Would help to have more of a back and forth with the person.

There are benefits that come from a voice-to-voice conversation that cannot be reproduced by typing into a prompt box.

##### Subtheme: Psychological challenges

Challenges with participants’ mental health and executive functions, such as stress, time management, organization, and problems with memory, impacted individuals’ ability to complete the e-CBT program.

Remembering when things needed to get done and setting time aside to do it.

Some, like myself, do not always understand terms or wording on certain subjects.

Difficulty staying on track during stressful life events.

## Discussion

This study explored patient perceptions of an AI-assisted triage system and a digital psychotherapy program implemented in a real-world psychiatric outpatient setting. Participants expressed both optimism and caution regarding the use of AI in mental healthcare. While many viewed the AI triage system as an effective tool for improving access to care, others raised concerns about the limitations of AI in capturing human complexity. The digital psychotherapy program received similarly mixed feedback, with some participants appreciating its flexibility and structure, while others encountered usability challenges or lacked sufficient human support. Thematic analysis was conducted using a reflexive approach, which prioritizes the depth, nuance, and contextual meaning of participant narratives over the frequency of theme occurrence. Therefore, we do not report the number of participants per theme or include a summary table, as this could imply a level of quantification inconsistent with our analytic aims.

Consistent with prior literature on AI in healthcare, our findings highlight the importance of trust and transparency in AI-based healthcare systems. Despite there being some support for the use of an AI-assisted triage system, due to previous difficulties in accessing traditional services, many participants expressed concerns with the use of AI for triaging. However, many clients also expressed openness to the use of AI during the triage process if it enabled faster access to mental health services, provided a clear boundary against AI replacing the therapeutic alliance in psychotherapy was maintained. Several participants expressed uncertainty about the AI's capabilities and emphasized the need for human oversight during the triage process. These findings align with established conceptualizations of DTA. Berry et al. (2020) propose that, in guided internet interventions, the alliance comprises task, goal, and bond components similar to face-to-face therapy, but these are mediated through the design and delivery of the digital platform.^19^ Sucala et al. (2012) further emphasize that the perceived relational quality in digital interventions is shaped by the responsiveness, personalization, and communication style of both the human and technological elements.^29^

Empirical studies have shown that higher levels of DTA are associated with greater engagement and lower dropout rates in digital psychotherapy programs.^21^ In our study, participants’ emphasis on personalization and human contact suggests that fostering a strong DTA, through both relational touchpoints and adaptive system design, may be critical for sustaining adherence and optimizing outcomes in AI-augmented care pathways.

This aligns with studies underscoring patient preference for clinician-led decision-making, even in digitally augmented settings.^30,31^

Some participants misunderstood the role of AI, believing it extended into diagnosis or treatment. This highlights a critical implementation challenge: End-users must be informed about the scope, limitations, and decision-making processes of AI tools. These insights carry practical implications for real-world deployment. Feedback from users suggests that system design should prioritize transparency, with clear delineation between AI-driven triage and human-led care. Additionally, communication strategies should be implemented to reduce confusion and build trust. As digital triage tools become more common, integrating user feedback during iterative development cycles may be key to achieving sustainable adoption at scale. The confusion expressed by some participants regarding the AI's role highlights the importance of providing clear and accessible information about how AI is used in clinical pathways. Misunderstandings about whether AI was involved in diagnosis or treatment suggest that future implementations should include educational materials, such as onboarding modules or brief infographics, to clarify that the AI functions only as a triage support tool, not as a replacement for clinician judgment. Therefore, the creation of resources to provide clients with a full understanding of the specific use of AI within the triage process could help clients better understand the use of the system.

Similarly, participants had varying experiences with the digital psychotherapy program. One of the main barriers to the program that likely increased participants’ desire to discontinue was technological difficulties. Therefore, further revision of the program to make it more user-friendly and intuitive is important. This could also include implementing more accessible features, such as speech-to-text. Furthermore, some participants found the program to be repetitive and therefore, psychoeducation could be used at the beginning of the modules to explain the reason for some of the repetition. In addition to general usability concerns, some feedback also pointed to potential challenges faced by neurodivergent users, suggesting a need for greater flexibility and inclusivity in program design. Although not a dominant theme, the concerns raised by neurodivergent participants highlight the importance of a more inclusive and accessible digital design. Incorporating more flexible, customizable, and neurodiversity-informed features could be an important modification to accommodate a broader range of user needs. Overall, people seemed to enjoy their care providers’ feedback since they found it to be helpful and reassuring. However, many participants commented on how increasing human contact would be beneficial since the people who had weekly calls found that this element kept them going throughout the program. However, when implementing more human contact, it is important that it does not only remain behind a screen and that there is voice-to-voice communication. This could include having an introductory call and a final call once the individual has finished all the digital psychotherapy sessions. Furthermore, the digital psychotherapy program currently sends out reminders to clients when their session due date is approaching. However, further helping individuals with executive functioning, such as organization and staying on top of their sessions, could help allow them to be successful and have a smooth journey within the program.

## Limitations and future directions

One of the limitations of this study is selection bias since participants could only be recruited from people who previously participated in the corresponding study and were from a single-site outpatient psychiatry waitlist. However, all of these clients actively engaged in the AI system and were capable of commenting on the program's efficacy. It would be ideal to conduct more studies on the AI system in different populations and use a larger sample. While the context-specific recruitment enhances the ecological validity of the findings, the broader external validity may be limited. These findings should inform future multisite implementation efforts with broader demographic representation in larger sample sizes. Additionally, there is also potential for self-selection bias since the survey was optional, and people who chose to do it might have been the ones who were more open to sharing their experience. It is possible that individuals with stronger opinions, either positive or negative, were more likely to respond, potentially skewing the thematic landscape. While the responses reflected a diversity of perspectives, including both supportive and critical views, the voices of participants with more neutral or disengaged experiences may be underrepresented. This limitation is common in qualitative research relying on open survey responses and highlights the need for future studies to use purposive sampling or targeted outreach to ensure that a broader range of perspectives is captured. Participants also experienced different levels of care within the digital psychotherapy program, such as receiving weekly phone calls, receiving only eCBT, or, for some, seeing a psychiatrist. This could have impacted individuals’ differing perceptions about the quality of care that they were provided. Furthermore, a notable limitation is the absence of demographic data (e.g. age, gender, education levels, digital literacy levels, etc.), which are known to influence perceptions of AI and digital health tools.^30–32^ Without this information, we were unable to analyze how perceptions may differ across subgroups, limiting the depth and nuance of our findings. Future research should incorporate demographic data to allow for more nuanced analysis of how user characteristics shape digital mental health experiences.

The attrition rate in this study (>50%) aligns with prior reports in digital mental health programs, where dropout rates of 30–80% are common.^33–35^ Contributing factors likely included technical issues, perceived repetitiveness, and the absence of real-time face-to-face human interaction. Furthermore, mental health symptoms such as low motivation or difficulty with organization, particularly in this real-world outpatient context without structured retention strategies likely contributed to attrition as well.

In addition to the aforementioned limitations, it was also found that there was a lack of clarity for patient participants regarding the extent to which the AI was involved in their care. Although the overall program incorporated both an AI-assisted triage and an eCBT program, the significant overlap between the two processes may have impacted participant perceptions when reflecting on their experiences. Due to this confusion, it is important to acknowledge the potential limitations in the data analysis conducted, as many participants’ experiences may or may not have been completely representative of their true opinions.

Future studies should investigate the best ways to improve communication between caregivers and patients around the use of AI-assisted tools, along with the benefits and implications of it. Additionally, future research utilizing survey data should incorporate optional low-burden demographic questions assessing variables such as categorical ranges for age, education levels, cultural backgrounds, and digital literacy. Collecting this data would enable more nuanced analyses of how user characteristics influence perceptions of AI-assisted mental health interventions and could guide the tailoring of such tools to improve accessibility and engagement across diverse populations. Other variables such as the specific levels of care that clients were provided with, may also be important considerations in assessing patient perceptions and experiences with similar programs.

In conclusion, this study examined participants’ perceptions of an AI-assisted triage system and a digital psychotherapy program. Responses were mixed for the use of an AI-assisted triage system and emphasized the importance of human oversight and personalization to ensure accuracy and meet the individual needs of clients. Participants also expressed appreciation for the use of AI in triaging and the ability to access care at an expedited rate. Furthermore, participants expressed various ways that digital psychotherapy programs could be improved, including making them more accessible for neurodiversity, improving the user-friendliness of the platform, and doing more psychoeducation on the reasons behind aspects like repetition. They also shared their enjoyment of the ability to receive care as they waited to see a psychiatrist and enjoyed the accessibility of the program.

## Conclusions

Patient experiences with AI-assisted triage and digital psychotherapy reflect a complex interplay of trust, usability, and perceived support. While many appreciated the increased access and timely care, others emphasized the enduring value of human oversight and connection. Participants’ experiences with the digital psychotherapy platform further emphasized the value of therapeutic support, usability, and the impact of technological barriers. These findings underscore the need for inclusive, transparent, and human-centered design when implementing AI in clinical mental health services. Future work should prioritize co-design with diverse users and ongoing evaluation to improve engagement and equity in digital care.

## Supplemental Material

sj-pdf-1-dhj-10.1177_20552076251384835 - Supplemental material for Perceptions and insights: A qualitative assessment of an AI-assisted psychiatric triage system implemented in an outpatient hospital setting Supplemental material, sj-pdf-1-dhj-10.1177_20552076251384835 for Perceptions and insights: A qualitative assessment of an AI-assisted psychiatric triage system implemented in an outpatient hospital setting by Oleksandr Knyahnytskyi, Jazmin Eadie, Kimia Asadpour, Callum Stephenson, Megan Yang, Taras Reshetukha, Christina Moi, Tricia Barrett, Meghanne Hicks, Gilmar Gutierrez-Nunez, Anchan Kumar, Jasleen Jagayat, Saad Sajid, Charmy Patel, Christina Holmes, Amir Shirazi, Vedat Verter, Claudio Soares, Mohsen Omrani and Nazanin Alavi in DIGITAL HEALTH

sj-pdf-2-dhj-10.1177_20552076251384835 - Supplemental material for Perceptions and insights: A qualitative assessment of an AI-assisted psychiatric triage system implemented in an outpatient hospital setting Supplemental material, sj-pdf-2-dhj-10.1177_20552076251384835 for Perceptions and insights: A qualitative assessment of an AI-assisted psychiatric triage system implemented in an outpatient hospital setting by Oleksandr Knyahnytskyi, Jazmin Eadie, Kimia Asadpour, Callum Stephenson, Megan Yang, Taras Reshetukha, Christina Moi, Tricia Barrett, Meghanne Hicks, Gilmar Gutierrez-Nunez, Anchan Kumar, Jasleen Jagayat, Saad Sajid, Charmy Patel, Christina Holmes, Amir Shirazi, Vedat Verter, Claudio Soares, Mohsen Omrani and Nazanin Alavi in DIGITAL HEALTH

sj-docx-3-dhj-10.1177_20552076251384835 - Supplemental material for Perceptions and insights: A qualitative assessment of an AI-assisted psychiatric triage system implemented in an outpatient hospital setting Supplemental material, sj-docx-3-dhj-10.1177_20552076251384835 for Perceptions and insights: A qualitative assessment of an AI-assisted psychiatric triage system implemented in an outpatient hospital setting by Oleksandr Knyahnytskyi, Jazmin Eadie, Kimia Asadpour, Callum Stephenson, Megan Yang, Taras Reshetukha, Christina Moi, Tricia Barrett, Meghanne Hicks, Gilmar Gutierrez-Nunez, Anchan Kumar, Jasleen Jagayat, Saad Sajid, Charmy Patel, Christina Holmes, Amir Shirazi, Vedat Verter, Claudio Soares, Mohsen Omrani and Nazanin Alavi in DIGITAL HEALTH

## Abbreviations

AI: artificial intelligence
eCBT: electronic cognitive behavioral therapy
DTA: digital therapeutic alliance
HDH: Hotel Dieu Hospital
KHSC: Kingston Health Sciences Centre
ML: machine learning
NLP: natural language processing
OCI: Ontario Centre of Innovation
OPTT: online psychotherapy tool
PI: principal investigator
QUOPL: Queen's University Online Psychotherapy Lab
QI: Quality improvement
