# Supplementary material for: Perceptions and insights: A qualitative assessment of an AI-assisted psychiatric triage system implemented in an outpatient hospital setting
Source: Digit Health. 2025 Oct 15;11:20552076251384835. doi: 10.1177/20552076251384835 (PMC12536140; doi:10.1177/20552076251384835)
Supplement: sj-pdf-2-dhj-10.1177_20552076251384835 - Supplemental material for Perceptions and insights: A qualitative assessment of an AI-assisted psychiatric triage system implemented in an outpatient hospital setting [file sj-pdf-2-dhj-10.1177_20552076251384835.pdf]

Default Question Block

Which care services did you receive?

e-CBT Program

Weekly phone and/or video call check-ins

Psychiatric appointments

All of the above

None of the above

How many psychiatric appointments did you have?

How many psychotherapy sessions did you complete?

Please provide your agreeance level for the following statements:

	Strongly Disagree	Somewhat Disagree	Neutral	Somewhat Agree	Strongly Agree	Not Applicable
AI triage allowed me to quickly get connected with a mental health care professional	○	○	○	○	○	○
The OPTT platform was easy to use	○	○	○	○	○	○

	Strongly Disagree	Somewhat Disagree	Neutral	Somewhat Agree	Strongly Agree	Not Applicable
Completing the symptom questionnaires helped me track my progress through the program	○	○	○	○	○	○
I am likely to recommend OPTT to friends or family in the future	○	○	○	○	○	○

What are your current thoughts on AI in healthcare?

What benefits/challenges did you face during your participation in this program?

Compared to care you have previously received, how was this program similar and/or different?

How would you compare the speed at which you were connected to resources/care providers, when compared to care you've previously received?

What barriers did you face throughout your engagement with this program?

Please provide any thoughts you have about the technology, psychotherapy program, triage process, and/or general experiences throughout this implementation.

Thank you for completing the survey. If you would like to be entered into a draw to win a $20 Amazon Gift Card, please provide your email below. The winners will be contacted individually.

Powered by Qualtrics
