# Supplementary material for: Perceptions and insights: A qualitative assessment of an AI-assisted psychiatric triage system implemented in an outpatient hospital setting
Source: Digit Health. 2025 Oct 15;11:20552076251384835. doi: 10.1177/20552076251384835 (PMC12536140; doi:10.1177/20552076251384835)
Supplement: sj-docx-3-dhj-10.1177_20552076251384835 - Supplemental material for Perceptions and insights: A qualitative assessment of an AI-assisted psychiatric triage system implemented in an outpatient hospital setting [file sj-docx-3-dhj-10.1177_20552076251384835.docx]

**Supplementary Materials**

**
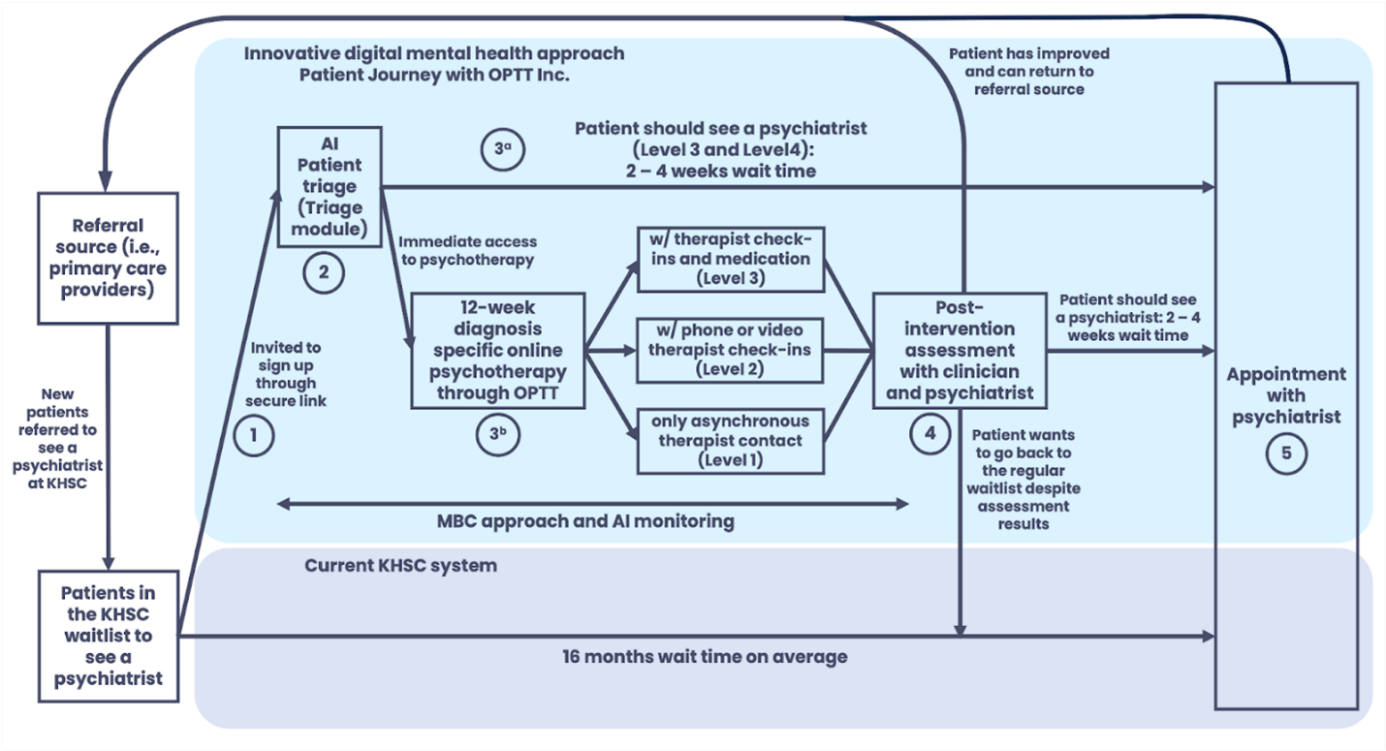
**

**Figure 1**

*Note.* Patient journey as part of the QI study conducted by our team at QUOPL. Figure outlines (1) the patient journey in the current triage system at KHSC and (2) the patient journey through the AI-triage approach.
